# Supplementary material for: The Implementation of Early Intervention Initiatives for Psychosis in Latin America and the Caribbean: A Case Study
Source: PLOS Glob Public Health. 2026 Mar 26;6(3):e0005531. doi: 10.1371/journal.pgph.0005531 (PMC13020790; doi:10.1371/journal.pgph.0005531)
Supplement: S1 Text — (DOCX) [file pgph.0005531.s002.docx]

**S1 Text: Interview guide**

**Please note that this is a guide and will be adjusted as needed to ensure natural flow of the conversation and to allow exploration of topics that are raised during the interview.**

Date:

Start Time:

End Time:

**Introduction**

Tell me a little about your work, and what you do? What does it involve?

How were you involved in implementing an initiative related to early psychosis?

**Exploration**

1. What do you think the health needs of people with early psychosis are? (Prompts: psychoeducation, psychotherapy, social support)
2. Why was this EIP intervention implemented (Prompts: evidence base, availability, training, cost)?
3. Was there any technical standard/guidelines in the country indicating the implementation of an EIP intervention for people with psychosis?
4. Were there other institutions that supported the idea of implementing the EIP intervention?
5. How important was leadership for the implementation of this intervention? Could you provide an example?
6. What was the capacities in early intervention in psychosis from mental health workers?
7. Could you describe the usual treatment of people with early psychosis in your country?
8. Was the development of this project conducted with the collaboration of any academic institutions?

**Preparation**

1. Did the EIP intervention receive support from health authorities? (Prompts: Ministry of Health, hospital management, NGO.)
2. Did service users show willingness or reluctance to participate in the EIP intervention?
3. How was the implementation of the EIP intervention financed?
4. Did the hospital's organizational system facilitate or hinder the implementation of the EIP intervention?
5. What resources did your team have at the beginning of the EIP intervention? Were they sufficient or not?
6. Did the health personnel show willingness or reluctance to implement the EIP intervention?
7. Did families of persons with psychosis show willingness or reluctance to participate in the EIP intervention?

**Implementation**

1. How has implementation of the EIP intervention been for service users? (change tense as needed; e.g., how was implementation if EIP initiative ended)
   1. Do you think service users believe in the EIP intervention?
2. How has implementation of the EIP intervention been for families? Do you think they believe in the EIP intervention?
3. Do you think the mental health professionals believe in the EIP intervention?
4. How is this intervention appropriate or not appropriate for service users? (Prompts: language, culture, needs, availability of services)
5. How is this intervention appropriate or not appropriate for your health facility?
6. Was any type of adaptation, such as cultural adaptation, made to the EIP intervention?

**Sustainment (These questions will be modified slightly for contexts like where there has been scaling up, e.g., how was the program funded to be scalable?)**

1. What are the options for sustaining or scaling up the EIP intervention in the future?
2. Is there any kind of advocacy for the rights of people with psychosis in your country (Prompts: Families associations)
3. How could the program be funded to achieve sustainability or be scalable?
4. Are there local or international partners who could support the sustainment or scaling up of the EIP intervention?
5. What resources would be needed to sustain or scaling up the EIP intervention? (Prompts: mental health resources, health facilities)
6. What changes would need to be made to the EIP intervention to sustain or scaling up it?
7. How do you think the academy could create greater capabilities in EIP?

Is there anything we have not discussed today about implementing services for the early stages of psychosis that would be important for us to know?

Do you have any ideas on how we can share the information from this study? With whom? What methods? For what purpose?
